# Mutation analysis of 419 family and prenatal diagnosis of 339 cases of spinal muscular atrophy in China

**DOI:** 10.1186/s12881-020-01069-z

**Published:** 2020-06-18

**Authors:** Yingjie Sun, Xiangdong Kong, Zhenhua Zhao, Xuechao Zhao

**Affiliations:** The Genetics and Prenatal Diagnosis Center, The First Affiliated Hospital of Zhengzhou University, Add: No. 1, Jianshe East Rd, Erqi District, Zhengzhou, Henan Province China

**Keywords:** Spinal muscular atrophy, SMN1 gene, MLPA, Long-range PCR, Prenatal diagnosis

## Abstract

**Background:**

Spinal muscular atrophy (SMA) is a common and lethal autosomal recessive neurodegenerative disease caused by mutations in the survival motor neuron 1 (SMN1) gene. At present, gene therapy medicine for SMA, i.e., Spinraza (Nusinersen), has been approved by the FDA, bringing hope to SMA patients and families. Accurate diagnosis is essential for treatment. Our goal was to detect genetic mutations in SMA patients in China and to show the results of the prenatal diagnosis of SMA.

**Methods:**

In this study, we examined 419 patients in our hospital from January 2010 to September 2019. Multiplex ligation-dependent probe amplification analysis was used to determine the copy numbers of SMN1 and SMN2. Long-range PCR combined with nested PCR was used to detect point mutations in SMN1. In addition to the above detection methods, we also used QF-PCR in prenatal diagnosis to reduce the impact of maternal contamination. We conducted a total of 339 prenatal diagnoses from January 2010 to September 2019.

**Results:**

Homozygous deletion of SMN1 exon 7 was detected in 96.40% (404/419) of patients. Homozygous deletion of SMN1 exon 7 alone was detected in 15 patients (3.60%). In total, 10 point mutations were detected in the 15 pedigrees. Most patients with SMA Type I have 1 ~ 2 copies of the SMN2 gene. Patients with SMA Type II have 2 or 3 copies of the SMN2 gene. The results of prenatal diagnoses showed that 118 fetuses were normal, 149 fetuses were carriers of heterozygous variants, and the remaining 72 fetuses harbored compound heterozygous variants or homozygous variants.

**Conclusions:**

Our study found that the most common mutation in SMA was homozygous deletion of SMN1 exon 7 in our study. We suggest that detecting only the deletion of exon 7 of SMN1 can meet most of the screening needs. We also believe that SMN2 copy numbers can help infer the disease classification and provide some reference for future treatment options.

## Background

Spinal muscular atrophy (SMA) is the most common autosomal recessive neuromuscular disease that causes death in infants and young children. The incidence of SMA is 1/10,000–1/6000, and the carrier rate is 1/50–1/38 [1]. Due to the degeneration of anterior horn motor neurons in the spinal cord, SMA patients have clinical manifestations of lower motor neuron injury, symmetrical proximal muscle weakness and muscle atrophy with the disappearance of tendon reflexes. Usually, the lower limbs are heavier than the upper limbs. According to the age of onset, maximum muscle activity and survival, SMA can be divided into four subtypes: infant type (SMAI) [OMIM 253300], intermediate type (SMAII) [OMIM 253500], juvenile type (SMAIII) [OMIM 253400] and adult type (SMAIV) [OMIM 271150] [2].

The pathogenic gene of SMA is the survival motor neuron (SMN) gene located on chromosome 5q13 [3]. This region has a complex structure with repeat sequences and many pseudo gene clusters, which make gene deletion and transformation easy. The SMN1 and SMN2 genes are two highly homologous genes and are arranged in tandem on the chromosome. SMN1 is also called SMNt because it is located on the telomere side, and SMN2 is also called SMNc because it is located on the centromere side [3]. The SMN1 gene is the causative gene for SMA, and the SMN2 gene functions to modify the SMA phenotype.

There are a total of five nucleotide differences between the two genes. They are mainly distinguished by two loci on exon 7 and exon 8. SMN1 is the main functional gene, and SMN1 deletion is the main cause of SMA [1].

At present, there are various methods for the genetic diagnosis of SMA, and these methods differ in terms of difficulty level, cost, stability, and experiment time. Common detection methods include fluorescent quantitative PCR (FQ-PCR), multiplex ligation-dependent probe amplification (MLPA) [4], PCR-restriction fragment length polymorphism (PCR-RFLP) [5], PCR-denaturing high-performance liquid chromatography (PCR-DHPLC), and long-range PCR [6].

MLPA is a successfully commercialized molecular diagnostic technique for SMA. The basic principle is to use multiple probes to target DNA sequences combined with capillary electrophoresis separation technology to achieve the diagnosis of SMA pathogenic genes. Since its development in 2002, this technology has been widely used clinically due to its high specificity, reliability, and stable commercial solutions [1, 7]. MLPA can perform accurate relative quantitative analysis of the SMN1 gene to distinguish between carriers, normal people and patients. At the same time, MLPA can also detect the copy number of SMN2, which is helpful to determine the subsequent treatment plan [8].

Approximately 95% of SMA cases are caused by deletion of exon of SMN1, and the MLPA method can be used for effective detection. However, for the remaining 5% of SMA cases caused by point mutations, MLPA cannot be used for effective detection. Since the SMN1 and SMN2 genes are highly homologous, the conventional NGS panel cannot distinguish whether the mutation is derived from the SMN1 gene or the SMN2 gene. In this circumstance, it is necessary to consider the combination of LR-PCR and nested PCR to solve the problem [9].

Since SMA is a severe/lethal autosomal recessive genetic disease, timely diagnosis of SMA is essential. In a well-diagnosed SMA family, the risk of recurrence for each child is 25% and is equal for males and females. For SMA families who still have fertility needs, prenatal genetic counseling and prenatal diagnosis are effective means to avoid giving birth to a sick child.

In this study, MLPA combined with LR-PCR, nested PCR, and Sanger sequencing was used for the genetic diagnosis of suspected SMA patients. For SMA families with fertility needs, SMA prenatal diagnosis was performed using MLPA combined with LR-PCR, nested PCR, Sanger sequencing, and QF-PCR.

## Methods

### Materials

A total of 419 patients with SMA from the Genetics and Prenatal Diagnostic Center of the First Affiliated Hospital of Zhengzhou University from January 2010 to July 2019 were enrolled in the study. Among them, 235 were male, and 184 were female. The age of treatment ranged from 45 days to 36 years. The clinical types and proportions of all patients are shown in Table 1. There were 177 patients with SMAI, 126 patients with SMAII, 100 patients with SMAIII and 16 patients with SMAIV. The SMN1 genes of the 419 SMA patients and their parents were analyzed. All SMA cases met the diagnostic criteria established by the International SMA Consortium (Munsat and Davies, 1992). The study was approved by the Ethics Committee of the First Affiliated Hospital of Zhengzhou University, and all subjects signed informed consent forms.

**Table 1:** Results of genetic diagnosis of SMA patients

Clinical type	Type I	Type II	Type III	Type IV	Total
Number	177	126	100	16	419
Proportion	45.6%	27.4%	23.2%	3.8%	100%

We suggested that pregnant women come to our hospital for villi extraction at 12–14 weeks of gestation for prenatal diagnosis. For pregnant women over 14 weeks of gestation, we recommend extraction of amniotic fluid at 16–20 weeks of gestation in our hospital for prenatal diagnosis. It is completely voluntary for any family to decide whether to take action based on the diagnosis. Overall, we conducted a total of 339 prenatal diagnoses from January 2010 to September 2019.

### DNA extraction

#### Genomic DNA extraction from human peripheral blood

Two milliliters of peripheral blood was taken from each proband and his/her parents and placed into an EDTA anticoagulation tube. Genomic DNA was extracted using OMEGA’s Mag-Bind® Blood & Tissue DNA HDQ 96 Kit. DNA concentration determination was performed using the Life Technologies Qubit dsDNA HS Assay Kit.

#### Genomic DNA extraction from chorionic villi

After informed consent was obtained, a small sample of chorionic villus tissue was taken from each pregnant woman by abdominal puncture under the guidance of B-ultrasound at 12–14 weeks of gestation. Intrauterine tissue was selected, and blood clots, placenta and other tissues were removed. Then, 3 to 4 pieces of villi of approximately 5 mm in length were selected and washed in normal saline. The villus samples were cut, and whole genomic DNA was extracted using a tissue extraction procedure of an Eppendorf epMotion 5075 m workstation (Eppendorf, Germany).

#### Genomic DNA extraction from amniotic fluid

After informed consent was obtained, 5 ml of amniotic fluid was taken from each pregnant woman by amniocentesis under B-ultrasound at 16 to 20 weeks of gestation. The genomic DNA was extracted immediately from amniotic fluid by using the German Qiagen Genomic DNA Extraction Kit, and the extracted DNA was stored in a low-temperature refrigerator at − 20 °C for subsequent use.

### MLPA testing

The extracted genomic DNA was diluted to 40 ng/μl, and 5 μl was taken and used for MLPA detection. SMN1 and SMN2 gene copy numbers were detected by using MLPA with the SALSA P060-B2 SMA Kit (MRC Holland) according to the manufacturer’s protocol. PCR products were analyzed on the ABI 3130 Genetic Analyzer (Applied Biosystems), and data were analyzed by Coffalyzer software (MRC Holland) or MLPA analysis–specific software (version 6.1, SoftGenetics, State College, PA). Ratios < 0.7, 0.7 < ratio < 1.3, 1.3 < ratio < 1.7, and 1.7 < ratio < 2.3 indicated one, two, three and four gene copies, respectively. All samples were analyzed at least twice.

### QF-PCR

Multiple QF-PCRs were performed on chromosomes 21, 18 and 13 and short tandem repeat (STR) sites on chromosomes. Chromosome 21 included 21A (D21 S1435), 21B (D21S11), 21C (D21S1411), 21D (D21S1444), 21H (D21S1442), and 21I (D21S1437). Chromosome 18 included 18B (D18 S978), 18C (D18 S535), 18D (D18S386), 18 J (D18S976), and 18 M (GATA178F11). Chromosome 13 included 13A (D13 S742), 13B (D13S634), 13C (D13S628), 13D (D13S305), and 13 K (D13S1492). The sex loci included X1 (DXS1187), X3 (XHPRT), X9 (DXS2390), SRY, XY2 (DXYS267), XY3 (DXYS218), AMELXY (AMELXY, AMELXY), ZFYX (ZFY, ZFX), T1 (indicating 7 and X chromosomes) and T3 (indicating 3 and X chromosomes) loci. The amplified product was subjected to capillary electrophoresis using an ABI-3130XL Genetic Analyzer, and GeneMapper accessory software v3 was used to analyze the presence of trisomy in the fetal chromosome. Maternal contamination was excluded.

### Long-range PCR

PCR was performed using KOD FX Neo Polymerase (TOYOBO, Osaka, Japan). The PCR product was detected by 1.5% agarose gel electrophoresis to confirm that the product length was as expected (see details in the supplementary materials).

### Nested PCR and sanger sequencing

Intragenic mutation and hybrid SMN gene analysis was performed by sequencing. We used 1 μl of purified long fragment PCR product as template to amplify each exon of SMN1 by nested PCR. Sequencing PCR primers and their annealing temperatures are listed in the supplementary materials. Two-step PCR was performed by using KOD FX polymerase (TOYOBO). Each SMN1 exon product was purified using the QIAquick PCR Purification Kit (Qiagen) and sequenced on the ABI 3130 Genetic Analyzer (Applied Biosystems) using the BigDye Terminator v3.1 Cycle Sequencing Kit. (see details in the supplementary materials.)

## Results

### Genetic diagnosis of SMA patients

#### SMN1 gene mutation analysis

**Fig. 1:**
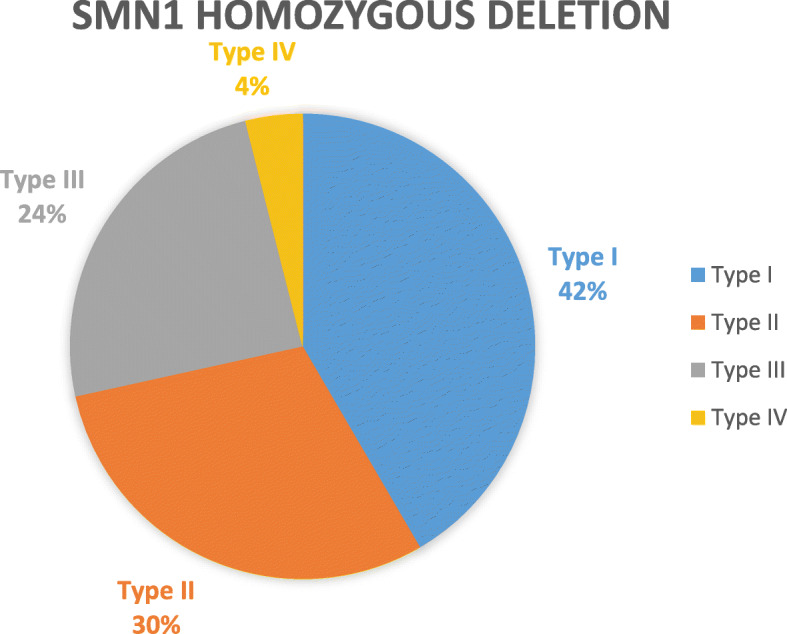
Different clinical phenotype distributions in SMN1 homozygous deletion patients

By using MLPA and long-range PCR, we found that approximately 96.4% (404/419) of patients had homozygous deletions for SMN1 (Table 2). Among these, 40.58% (168/414) of patients were diagnosed with SMA Type I, 29.23% (121/414) with Type II, 23.91% (99/414) with Type III, and 3.86% (16/414) with Type IV (Fig. 1).

**Table 2:** SMN1 copy numbers in patients with different clinical types of SMA

Clinical types	Type I	Type II	Type III	Type IV	Total
0	168 (40.10%)	121 (28.88%)	99 (23.63%)	16 (3.82%)	404 (96.42%)
≥1	9 (2.18%)	5 (1.19%)	1 (0.24%)	0 (0.00%)	15 (3.58%)
Total	177 (42.24%)	126 (30.07%)	100 (23.87%)	16 (3.82%)	419 (100.00%)

In the remaining 3.6% (15/419) of cases, heterozygous deletion of SMN1 E7 was associated with point mutation. The point mutation in patients was found to be inherited from their parents, and the de novo mutation rate was 0. The deletion rate of the SMN1 E7 allele was 98.21%, and the point mutation rate was 1.79%. A total of 10 point mutations were found in the 15 point mutation families. The specific information is listed in Table 3.

**Table 3:** Mutation information of SMA patients

Changes of cDNA	Exon	Novel
c.21_22insA	1	Y
c.22dupA	1	N
c.56delT	1	Y
c.683 T > A	5	N
c.689C > T	5	N
c.835G > C	7	Y
c.836G > T	7	N
c.863G > T	8	Y
c.840C > T	8	N
c.885 + 3A > T	8	Y

#### SMN2 gene copy number analysis

In 419 patients, the distribution of SMN2 copy number was as follows: one copy (11.46%), two copies (65.87%), three copies (19.57%), and four copies (3.10%). No homozygous deletion of SMN2 was observed. The copies of SMN2 in patients with different clinical subtypes of SMA are shown in Table 4.

**Table 4:** SMN2 copy numbers in patients with different clinical types of SMA

Clinical types	SMN2 copy number				Total
	1	2	3	4	
Type I	46 (10.98%)	126 (30.07%)	5 (1.19%)	0 (0.00%)	177 (42.24%)
Type II	2 (0.48%)	101 (24.11%)	19 (4.53%)	4 (0.95%)	126 (30.07%)
Type III	0 (0.00%)	47 (11.22%)	49 (11.70%)	4 (0.95%)	100 (23.87%)
Type IV	0 (0.00%)	2 (0.48%)	9 (2.15%)	5 (1.19%)	16 (3.82%)
Total	48 (11.46%)	276 (65.87%)	82 (19.57%)	13 (3.10%)	419

### Prenatal diagnosis of SMA family

In the current study, a total of 339 prenatal diagnoses were made for 293 families. Among them, 252 families underwent one prenatal diagnosis, 38 families underwent two prenatal diagnoses, and 3 families underwent three prenatal diagnoses. For one family, the three prenatal diagnoses corresponded to three singleton pregnancies. For one family, the three prenatal diagnoses corresponded to a twin pregnancy and a singleton pregnancy. The results showed that one fetus of the twin was normal and the other was an SMA fetus. After the fetus was reduced in the reproductive center, the remaining single fetus was verified as normal.

The prenatal diagnosis revealed 72 SMA cases (labor was induced) and 267 carriers and normal cases. Among the later cases, 3 were miscarriage cases due to other reasons, and the remaining 336 cases were followed up after birth without any missed diagnosis. The gender and genotype distribution of the prenatal diagnosis results are shown in Table 5.

**Table 5:** SMA prenatal diagnosis results

Results	Patient	Carrier	Normal person	Total
Male	41 (12.1%)	71 (20.9%)	52 (15.3%)	164 (48.1%)
Female	31 (9.1%)	78 (23.0%)	66 (19.5%)	175 (51.6%)
Total	72 (21.2%)	148 (43.9%)	118 (34.8%)	339 (100%)

## Discussion

We analyzed the SMN1 gene in 419 SMA patients, with a male to female ratio of 235:184. Among the patients with SMAII and SMAIII, the proportions of male patients were 56 and 53%, respectively. In 404 SMA patients, the 7th exon of the SMN1 gene was found to be homozygously deleted. In 88.6% (359/404) of the patients, homozygous deletion of exon 7 and heterozygous deletion of the 8th exon of the SMN1 gene were found. In 11.1% (45/404) of the patients, homozygous deletion of only the 7th exon of the SMN1 gene was found. This result is similar to previous studies. Studies have shown that 95% of SMA cases are caused by homozygous deletion of the 7th and 8th exons or only the 7th exon of the SMN1 gene, and approximately 5% of SMA cases are caused by complex heterozygous mutation of the SMN1 gene. We also found that the deletion rate of the SMN1 E7 allele was 98.21%, and the point mutation rate was 1.79%, which is similar to other research. The homozygous deletion rate of 419 SMA children was 96.4% (404/419), which was consistent with Chinese populations and international studies, indicating that the majority of SMA children had a homozygous deletion of the SMN1 gene, which made genetic analysis a clinical diagnosis. In the SMA children with homozygous deletion, no homozygous deletion of exon 8 alone was observed, suggesting that the analysis of homozygous deletion of exon 7 of the SMN1 gene is more diagnostic for children with SMA.

Researchers have used long-range PCR to find nearly 100 small mutations in the SMN gene, most of which are missense mutations and nonsense mutations [10]. A few small mutations are located at the splicing site, while most of the small mutations are located at the junction of exon 3 and exon 6 or the exon-intron junction of the SMN1 gene. It has been reported that 3 to 5% of SMA patients have small mutations in the SMN1 gene. In this study, heterozygous deletions of exons 7 and 8 of the SMN1 gene were detected in 3.6% of SMA patients.

SMA is a serious disease with a high carrier rate and a high incidence of disability and death, and its early symptoms are not obvious. Because the disease has no effective treatment, it brings heavy psychological and economic burdens to the family, so it is necessary to conduct carrier screening. In 2016, the American Society of Medical Genetics (ACMG) issued technical standards and laboratory guidelines for SMA testing. In 2017, the American College of Obstetricians and Gynecologists (ACOG) required all women who had children to be screened for SMA carriers. Carriers can be divided into two groups: those who have a mutation in one of the two alleles of the common SMN1 gene and those who carry two copies of the normal SMN1 gene, but both are in the same chromatid (which is known as “2 + 0”). In our study, we found that all the patients in this study had the deletion (homozygous deletion or heterozygous deletion combined with point mutation), and we also found that the mutation of the patients was inherited from their parents by carrier tests of their parents. Therefore, we believe that the detection of exon 7 can meet the needs of carrier screening in most cases. For “2 + 0” carriers, current technology cannot detect them. Considering the economic cost and other factors, we believe that carrier screening for exon 7 of SMN1 is the most cost-effective screening method.

We also detected the number of copies of SMN2. Although carrier screening has been shown to reduce the number of SMA births, researchers are still trying to find ways to treat children who already have SMA. With the continuous development of technology, some drugs have entered clinical trials and are even being marketed. The advanced biopharmaceutical company [11]. Cytokinetics was awarded CK-2127107 by the US Food and Drug Administration as an experimental drug for the treatment of spinal muscular atrophy (SMA). CK-2127107 is a new generation of fast-acting skeletal muscle troponin activator (FSTA) developed by Cell Dynamics and Japanese pharmaceutical companies, which is expected to treat spinal muscular atrophy (SMA) and amyotrophic spinal cord diseases related to skeletal muscle weakness and/or fatigue, such as sclerosis (ALS). Spinraza, developed by Boston biotechnology company Scholar Rock, works by upregulating the expression of SMN proteins in patients, and muscle drugs such as SRK-015 can be used in combination with them or on their own. SRK-015 combined with upregulated SMN expression drugs can increase muscle strength in SMA mice by 60%. Gene therapy is a promising treatment. It uses a viral vector that can carry normal SMN genes into the body without integrating into DNA molecules. The AAV9 virus vector can transfer the SMN1 gene to motor neurons, induce continuous and rapid expression of the SMN protein, and alleviate disease symptoms [12]. Spinraza is an antisense nucleotide that combines with the splicing site of SMN2 exon 7 to correct the RNA splicing of the defective SMN2 gene to produce a protein product that can better replace the function of the SMN1 gene, thereby achieving the role of disease treatment [13]. In our study, we found that SMN2 copy number was inversely correlated with disease severity, which is similar to previous international studies [14]. We found that some treatments modified the SMN2 gene to compensate for the impaired SMN protein synthesis caused by defects in the SMN1 gene. Therefore, detecting the normal copy number of SMN2 in patients is of great significance for this part of treatment [15].

## Conclusions

Our study found that the most common mutation in SMA was homozygous deletion of SMN1 exon 7 in our study. We suggest that detecting only the deletion of exon 7 of SMN1 can meet most of the screening needs. We also believe that SMN2 copy numbers can help infer the disease classification and provide some reference for future treatment options.

## Supplementary information

**Additional file 1.**

## Data Availability

The datasets generated and/or analysed during the current study are not publicly available because patient details were involved, the data needed to be kept confidential. The raw datasets are available from the corresponding author on reasonable request.

## Abbreviations

SMA: Spinal muscular atrophy
SMN: Survival motor neuron
FQ-PCR: Fluorescent quantitative PCR
MLPA: Multiplex ligation-dependent probe amplification
NGS: “Next-generation” Sequencing
AAV9: Adeno-associated Virus Type 9
