# Supplementary material for: Mutation analysis of 419 family and prenatal diagnosis of 339 cases of spinal muscular atrophy in China
Source: BMC Med Genet. 2020 Jun 18;21:133. doi: 10.1186/s12881-020-01069-z (PMC7302341; doi:10.1186/s12881-020-01069-z)
Supplement: Supplementary file 1 — Additional file 1. [file 12881_2020_1069_MOESM1_ESM.pdf]

Supplementary Materials:

1. Long-range PCR

The PCR system consisted of 25 μl of 2×PCR buffer, 10 μl (0.4 mM) of each of 4 dNTPs, 0.3 μl (0.15 mM) of each primer (Primer Fw and Primer Rv), 1 μl of polymerase and 0.4 μl of genomic DNA. Water was added to bring the volume of the reaction system to 50 μl. (Supplementary Table 1). The reaction conditions were set as follows: predenaturation at 94 ℃ for 2 min, then denaturation at 98 ℃ for 5 cycles for 10 s and annealing at 71.2 ℃ for 15 min; denaturation at 98 ℃ for 5 cycles for 10 s and annealing at 69.2 ℃ for 28 min; denaturation at 98 ℃ for 5 cycles for 10 s and annealing at 67.2 ℃ for 28 min; denaturation at 98 ℃ for 10 s and annealing at 65.2 ℃ for 28 min; and finally remaining at 65.2 ℃ for another 15 min.

Table 1.Experimental system

28 kb (50 μl)		13.2 kb (25 μl)	
2X Buffer:	25 μl	2X Buffer:	12.5 μl
dNTP:	10 μl	dNTP:	5 μl
KOD:	1 μl	KOD:	1 μl
DNA:	0.4 μl	DNA:	0.4 μl
Primer Fw:	0.3 μl	Primer Fw:	0.3 μl
Primer Rv:	0.3 μl	Primer Rv:	0.3 μl
dH2O:	13 μl	dH2O:	5.5 μl
50 μl		25 μl	

Experimental conditions for 28 kb: (Step down 71.2—65.2):

94 ℃	2 min	
98 ℃	10 s	5 cvcles
71.2 ℃	15 min	
98 ℃	10 s	5 cvcles
69.2 ℃	28 min	

98 ℃	10 s	5 cvcles
67.2 ℃	28 min	
98 ℃	10 s	8 cvcles
65.2 ℃	28 min	
65.2 ℃	15 min	
4 ℃	Forever	

Experimental condition for 31.2 kb:

94 ℃	2 min	
98 ℃	10s	32 cvcles
63 ℃	30 s	
68 ℃	14 min	
68 ℃	10 min	
4 ℃	∞	

2. Nested PCR and Sanger sequencing

The reaction system consisted of 12.5 μl of 2× PCR buffer, 5 μl (0.4 mM) of each dNTP, 0.3 μl (0.4 mM) of each primer, 1 μl of polymerase, 0.4 μl of template and water (Supplementary Table 1). PCR was carried out under the following conditions: 94 ℃ for 2 min, then denaturation at 98 ℃ for 35 cycles for 10 s, and then annealing at 68 ℃ for 45 s. For amplification of other targets, the PCR system consisted of 2.5 μl of 10×Ex Taq buffer, 0.2 mM of each dNTP, 0.4 μM of each primer, 1.25 U of polymerase, template and water (Supplementary Table 1). PCR was carried out under the following conditions: initial denaturation at 94 ℃ for 2 min, then denaturation at 98 ℃ for 32 cycles for 10 s, annealing at 63 ℃ for 30 s, extension at 68 ℃ for 14 min, and finally extension at 68 ℃ for 5 min.

Experimental system:

2x Taq PCR Master Mix:	12.5 μl
F+R(E7/E8):	0.4 μl
DNA (50 ng/μl)：	0.4 μl
dH2O:	11.7 μl
Total	25 μl

Experimental conditions:

95 ℃	4 min	
95 ℃	30 s	32 cvcles
60/68 ℃	30 s	
72 ℃	30 s	
72 ℃	7 min	
4 ℃	∞	

Table 2. Primer sequences used for long-range PCR and nested PCR

Target Region	Primer Sequence (5'--3’)	Length (bp)	Tm (℃)
Ex1~8(FL-Fv)	GTTGGGGGATCAAATATCTTCTAGTGTT	28233	Step down
Ex1~8(FL-Rv)	CCCCCACCCCAGTCTTTTACAGATGGT		71.2--65.2
Ex2a-E7-FL-Fw	TGTGTGGATTAAGATGACTC	13.2k	60
Ex2a-E7-FL-Rv	CCTTCCTTCTTTTTGATTTTGTCTG		
Ex1-Fw	GCGAGGCTCTGTCTCAAAACA	439	68
Ex1-Rv	GATCGACTTGATGCTGTCCCGA		
Ex2a-Fw	CACATAACCTCTAACCAGGTAA	396	60
Ex2a-Rv	GGAGGATATCACCTGATTTAACT		
Ex2b-Fw	GGTGTATGATGCCTTTAAGAGCAGTTT	555	60
Ex2b-Rv	CTTCTCCCTGCCTTCCATTCACA		
Ex3-Fw	GCACCATACGCATTTTATCTC	690	60
Ex3-Rv	GAAACTTGGCTTTCATTTTCATTC		
Ex4-Fw	TTCAATTTCTGGAAGCAGAGA	383	60
Ex4-Rv	CAAAAGTTTCATGGGAGAGC		
Ex5-Fw	GACTTCAGGATTTGGTACATGA	354	60
Ex5-Rv	CCCAAGGGATGTTCTACAATGAC		
Ex6-Fw	CAACATAGCAAGACCTCGTCT	431	60
Ex6-Rv	TGCAAGAGTAATTTAAGCCTCAGA		

Ex7-Fw	GCTCCAGGTCTCAAGTGAT	680	60
Ex7-Rv	GTGCAGTATGCCTAGGTTAT		
